# Supplementary material for: Robust estimation of heritability and predictive accuracy in plant breeding: evaluation using simulation and empirical data
Source: BMC Genomics. 2020 Jan 14;21:43. doi: 10.1186/s12864-019-6429-z (PMC6958597; doi:10.1186/s12864-019-6429-z)
Supplement: Supplementary file 1 — Additional file 1 This file contains additional Tables S1,...,S7. First- and second-stage results are reported together. [file 12864_2019_6429_MOESM1_ESM.pdf]

Appendix A
Supplementary tables

Table S1. Mean squared deviations of the estimated genotypic means from the true breeding values – $\text{MSD}_\mu = \sum_{i=1}^{698} \sum_{l=1}^{1000} \frac{(\widehat{\mu}_{il} - \mu_{il})^2}{698 \times 1000}$ – and mean squared deviations of the estimated breeding values from the true breeding values – $\text{MSD}_g = \sum_{i=1}^{698} \sum_{l=1}^{1000} \frac{(\widehat{g}_{il} - g_{il})^2}{698 \times 1000}$ – for the classical (CLS) and robust (ROB) methods under the **random** contamination scenarios. MSDs of the classical from the robust estimates are also reported (CLS-ROB).

**Scenarios I**		**1st-stage ($\widehat{\boldsymbol{\mu}}$)**			**2nd-stage ($\widehat{\mathbf{g}}$)**		
% cont	sd	**CLS**	**ROB**	**CLS-ROB**	**CLS**	**ROB**	**CLS-ROB**
0	-	28.97	29.08	0.06	25.18	25.48	0.05
1	5	32.44	32.51	0.09	26.29	26.26	0.13
1	8	37.83	37.78	0.15	27.84	26.44	0.54
1	10	42.80	42.64	0.21	29.16	26.49	1.01
3	5	39.36	39.43	0.13	28.16	27.66	0.23
3	8	55.46	55.27	0.24	31.95	28.30	1.05
3	10	70.23	69.89	0.33	34.88	28.48	1.96
5	5	46.26	46.39	0.18	29.37	28.72	0.27
5	8	72.98	72.87	0.27	34.53	29.97	1.18
5	10	97.50	97.25	0.33	38.31	30.33	2.25
7	5	53.12	53.34	0.24	31.35	30.59	0.27
7	8	90.42	90.44	0.31	37.99	32.92	1.07
7	10	124.68	124.55	0.34	42.59	33.57	2.07
10	5	63.30	63.65	0.31	32.53	32.41	0.25
10	8	116.40	116.67	0.55	40.39	37.40	0.73
10	10	165.22	165.30	0.58	45.71	39.04	1.28

% `cont` stands for the percentage of contamination; `sd` stands for the number of standard deviations of the outliers.

Table S2. The overall mean value (om) of the estimated genotypic means ($\widehat{\boldsymbol{\mu}}$) and breeding values ($\widehat{\mathbf{g}}$) together with the corresponding Pearson correlation coefficients (r_p) between the estimates of $\widehat{\boldsymbol{\mu}}$ and $\widehat{\mathbf{g}}$ and the true breeding values, obtained using the classical (CLS) and the robust (ROB) methods under the **random** contamination scenarios. The true overall genotypic mean is 8.923 (computed as the average of the true 698 breeding values $\mathbf{g}$).

**Scenarios I**		**1st-stage ($\widehat{\boldsymbol{\mu}}$)**		**2nd-stage ($\widehat{\mathbf{g}}$)**	
% cont	sd	**CLS** (om/r_p)	**ROB** (om/r_p)	**CLS** (om/r_p)	**ROB** (om/r_p)
0	-	8.908/0.76	8.906/0.76	5.001/0.90	4.935/0.90
1	5	9.092/0.75	9.090/0.75	4.925/0.90	4.880/0.90
1	8	9.203/0.72	9.200/0.72	4.823/0.89	4.861/0.90
1	10	9.276/0.70	9.274/0.70	4.737/0.88	4.856/0.90
3	5	9.460/0.71	9.458/0.71	4.822/0.89	4.791/0.89
3	8	9.792/0.65	9.789/0.65	4.586/0.86	4.729/0.89
3	10	10.013/0.61	10.010/0.61	4.405/0.85	4.713/0.89
5	5	9.829/0.69	9.827/0.69	4.758/0.88	4.730/0.89
5	8	10.381/0.60	10.379/0.60	4.442/0.85	4.612/0.88
5	10	10.749/0.55	10.747/0.55	4.214/0.82	4.577/0.88
7	5	10.196/0.67	10.194/0.66	4.599/0.87	4.576/0.88
7	8	10.969/0.57	10.967/0.57	4.189/0.83	4.374/0.87
7	10	11.484/0.51	11.483/0.51	3.918/0.80	4.315/0.87
10	5	10.748/0.64	10.748/0.63	4.562/0.86	4.472/0.87
10	8	11.853/0.52	11.853/0.52	4.092/0.81	4.0787/0.85
10	10	12.589/0.46	12.589/0.46	3.789/0.78	3.948/0.84

% `cont` stands for the percentage of contamination; `sd` stands for the number of standard deviations of the outliers.

Table S3. Mean squared deviations of the estimated genotypic means from the true breeding values – $\text{MSD}_\mu = \sum_{i=1}^{698}\sum_{l=1}^{1000} \frac{(\widehat{\mu}_{il} - \mu_{il})^2}{698 \times 1000}$ – and mean squared deviations of the estimated breeding values from the true breeding values – $\text{MSD}_g = \sum_{i=1}^{698}\sum_{l=1}^{1000} \frac{(\widehat{g}_{il} - g_{il})^2}{698 \times 1000}$ – for the classical (CLS) and robust (ROB) methods under the **block** contamination scenarios. MSDs of the classical from the robust estimates are also reported (CLS-ROB).

**Scenarios II**		**1st-stage ($\widehat{\boldsymbol{\mu}}$)**			**2nd-stage ($\widehat{\mathbf{g}}$)**		
No. blocks	sd	**CLS**	**ROB**	**CLS-ROB**	**CLS**	**ROB**	**CLS-ROB**
0	-	28.97	29.08	0.06	25.18	25.55	0.05
1	5	30.90	29.45	0.91	25.35	25.61	0.19
1	8	30.75	29.69	1.76	25.38	25.63	0.27
1	10	31.16	29.83	2.31	25.39	25.63	0.30
2	5	30.93	29.53	1.41	25.32	25.56	0.21
2	8	32.49	29.81	3.08	25.36	25.58	0.28
2	10	33.79	29.93	4.41	25.37	25.59	0.31
3	5	32.06	29.64	2.06	25.34	25.65	0.22
3	8	35.22	29.96	5.08	25.37	25.68	0.29
3	10	38.02	30.11	7.74	25.37	25.67	0.30
4	5	33.48	29.56	2.70	25.34	25.54	0.18
4	8	38.74	29.61	7.25	25.37	25.54	0.24
4	10	43.51	29.63	11.52	25.37	25.54	0.25
5	5	35.31	29.76	3.69	25.39	25.58	0.17
5	8	43.33	29.83	10.51	25.40	25.58	0.22
5	10	50.66	29.88	17.03	25.40	25.59	0.24

`No Blocks` stands for the number of contaminated blocks; `sd` stands for the number of standard deviations of the outliers.

Table S4. The overall mean value (om) of the estimated genotypic means ($\widehat{\boldsymbol{\mu}}$) and breeding values ($\widehat{\mathbf{g}}$) together with the corresponding Pearson correlation coefficients (r_p) between the estimates of $\widehat{\boldsymbol{\mu}}$ and $\widehat{\mathbf{g}}$ and the true breeding values, obtained using the classical (CLS) and the robust (ROB) methods under the **block** contamination scenarios. The true overall genotypic mean is 8.923 (computed as the average of the true 698 breeding values $\mathbf{g}$).

**Scenarios II**		**1st-stage ($\widehat{\boldsymbol{\mu}}$)**		**2nd-stage ($\widehat{\mathbf{g}}$)**	
No. blocks	sd	**CLS** (om/r_p)	**ROB** (om/r_p)	**CLS** (om/r_p)	**ROB** (om/r_p)
0	-	8.908/0.77	8.906/0.76	4.979/0.90	4.935/0.90
1	5	9.379/0.76	9.017/0.76	5.004/0.90	4.933/0.90
1	8	9.661/0.76	9.022/0.76	5.008/0.90	4.930/0.90
1	10	9.849/0.76	9.025/0.76	5.009/0.90	4.929/0.90
2	5	9.837/0.76	9.112/0.76	5.012/0.90	4.939/0.90
2	8	10.394/0.76	9.119/0.76	5.013/0.90	4.936/0.90
2	10	10.765/0.76	9.121/0.76	5.014/0.90	4.934/0.90
3	5	10.289/0.76	9.211/0.76	5.011/0.90	4.932/0.90
3	8	11.118/0.76	9.218/0.76	5.013/0.90	4.929/0.90
3	10	11.670/0.76	9.220/0.76	5.013/0.90	4.929/0.90
4	5	10.715/0.76	9.299/0.76	5.015/0.90	4.946/0.90
4	8	11.799/0.76	9.300/0.76	5.015/0.90	4.946/0.90
4	10	12.522/0.76	9.301/0.76	5.015/0.90	4.946/0.90
5	5	11.153/0.76	9.411/0.76	5.008/0.90	4.942/0.90
5	8	12.500/0.76	9.413/0.76	5.011/0.90	4.941/0.90
5	10	13.398/0.76	9.414/0.76	5.011/0.90	4.941/0.90

`No Blocks` stands for the number of contaminated blocks; `sd` stands for the number of standard deviations of the outliers.

Table S5. Mean squared deviations of the estimated breeding values and the true breeding values ($MSD_{\mathbf{g}}$) for the classical (CLS) approach computed using the Smith's and standard weights for the **block** and **random** contamination scenarios.

**Block scenarios**		**Smith**	**Standard**	**Random scenarios**		**Smith**	**Standard**
No. blocks	sd			% cont	sd		
0	-	25.18	25.29	0	-	25.18	25.29
1	5	25.35	25.61	1	5	26.29	26.40
1	8	25.38	25.77	1	8	27.85	27.96
1	10	25.39	25.87	1	10	29.16	29.28
2	5	25.33	25.74	3	5	28.16	28.31
2	8	25.36	26.09	3	8	31.95	32.16
2	10	25.37	26.40	3	10	34.88	35.13
3	5	25.34	25.99	5	5	29.38	29.61
3	8	25.37	26.75	5	8	34.53	34.93
3	10	25.37	27.49	5	10	38.31	38.85
4	5	25.34	26.31	7	5	31.35	31.70
4	8	25.37	27.70	7	8	37.99	38.64
4	10	25.37	29.08	7	10	42.59	43.48
5	5	25.39	26.80	10	5	32.53	33.20
5	8	25.40	29.05	10	8	40.39	41.73
5	10	25.40	31.27	10	10	45.71	47.56

% `cont` stands for percentage of contamination; `sd` stands for the number of standard deviations of the outliers;

`No Blocks` stands for the number of contaminated blocks.

Table S6. The mean squared deviation of heritability – $\text{MSD}_{\texttt{H}} = \sum_{l=1}^{1000} \frac{(\widehat{H}_l^2 - (r_{g,\widehat{g},l})^2)^2}{1000}$ (Method 5) – and predictive accuracy – $\text{MSD}_{\texttt{PA}} = \sum_{l=1}^{1000} \frac{(\widehat{r}_{g,\widehat{g},l} - r_{g,\widehat{g},l})^2}{1000}$ (Methods 5 and 7) – of the classical (CLS) from the robust (ROB) method under the **random** contamination scenarios.

**Random scenarios**		$\texttt{H}^2$ – **M5**		$\texttt{PA}$ – **M5**		$\texttt{PA}$ – **M7**	
% cont	sd	**CLS**	**ROB**	**CLS**	**ROB**	**CLS**	**ROB**
0	-	0.01	0.01	0.00	0.00	0.01	0.01
1	5	0.02	0.01	0.00	0.00	0.01	0.01
1	8	0.02	0.01	0.00	0.00	0.01	0.01
1	10	0.02	0.01	0.00	0.00	0.01	0.01
3	5	0.02	0.02	0.00	0.00	0.01	0.01
3	8	0.03	0.02	0.00	0.00	0.01	0.01
3	10	0.03	0.02	0.00	0.00	0.02	0.01
5	5	0.02	0.02	0.00	0.00	0.01	0.01
5	8	0.04	0.02	0.00	0.00	0.02	0.02
5	10	0.06	0.02	0.01	0.00	0.02	0.02
7	5	0.03	0.03	0.00	0.00	0.01	0.02
7	8	0.05	0.03	0.01	0.00	0.02	0.03
7	10	0.07	0.04	0.01	0.00	0.03	0.03
10	5	0.03	0.03	0.00	0.00	0.02	0.03
10	8	0.07	0.06	0.01	0.01	0.03	0.06
10	10	0.09	0.07	0.02	0.01	0.04	0.07

% `cont` stands for percentage of contamination; `sd` stands for the number of standard deviations of the outliers.

Table S7. Summary table of comparative perfomance of the competing methods for the block and random contamination scenarios; performance for the NULL scenarios was similar and is thus not shown.

**Method**	**Scenario**	**1st-stage**					**2st-stage**			**3rd-stage**	
		$\mathbf{MSD}_\mu$	r_p	σ_r^2	$\sigma_{r:b}^2$	σ_e^2	$\mathbf{MSD}_g$	r_p	σ_s^2	H^2	PA
CLS	random	s	s	w	b	w	w	w	w	w	w
	block	w	s	w	w	s	s	s	w	s	s
ROB	random	s	s	b	w	b	b	b	b	b	b
	block	b	s	b	b	s	s	s	b	s	s

"b" means *better* and "w" means *worse* performance than the competing method;

"s" means *similar* performance as the competing method.
