# Supplementary material for: Robust estimation of heritability and predictive accuracy in plant breeding: evaluation using simulation and empirical data
Source: BMC Genomics. 2020 Jan 14;21:43. doi: 10.1186/s12864-019-6429-z (PMC6958597; doi:10.1186/s12864-019-6429-z)
Supplement: Supplementary file 2 — Additional file 2 This file contains additional Figures S1,...,S20. Results reported in these figures are organized from the first stage to the third stage. [file 12864_2019_6429_MOESM2_ESM.pdf]

Appendix B

Supplementary figures

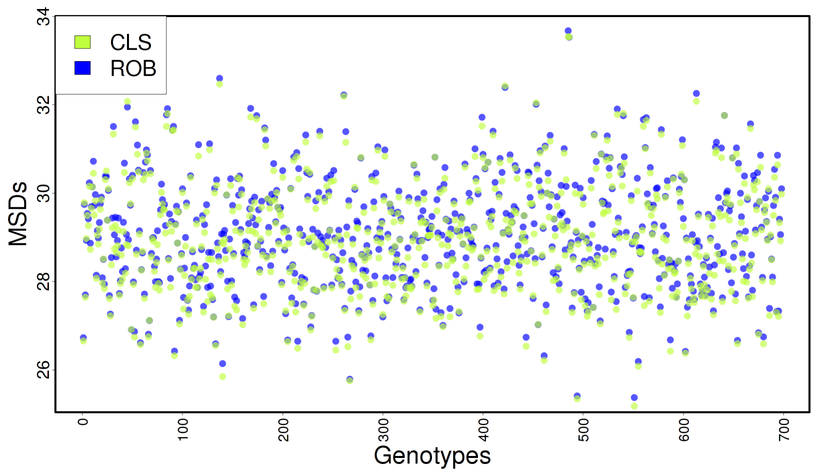

Figure S1. Plot of the classical and robust $\text{MSD}_{\mu}^{i} = \sum_{l=1}^{1000} \frac{(\widehat{\mu}_{il} - \mu_{il})^2}{1000}$ for each of the 698 genotypes for the **null** scenario (`1st stage`)

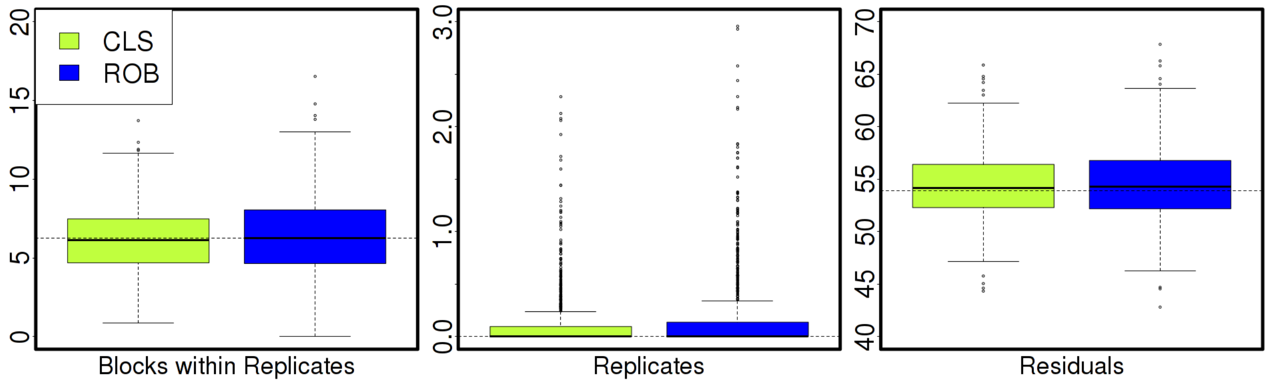

Figure S2. Boxplots of the 1000 classical and robust estimated *block* ($\sigma^2_{r:b}$), *replicate* (σ^2_r) and *residual* variances (σ^2_e) for the **null** scenario (`1st stage`)

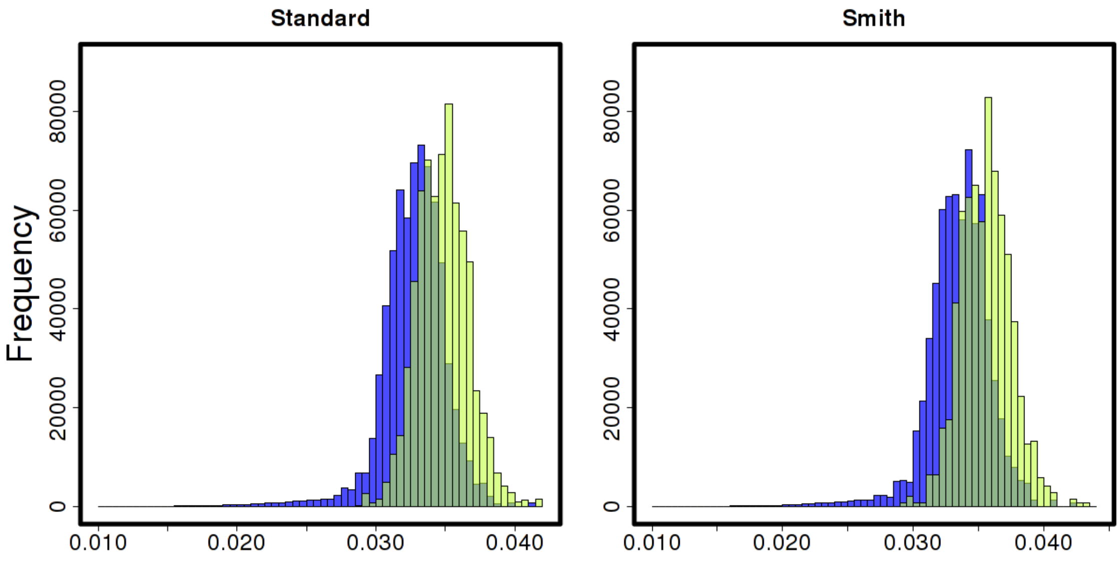

Figure S3: Histograms of the classical and robust Standard and Smith's weights for the **null** scenario (`1st stage`)

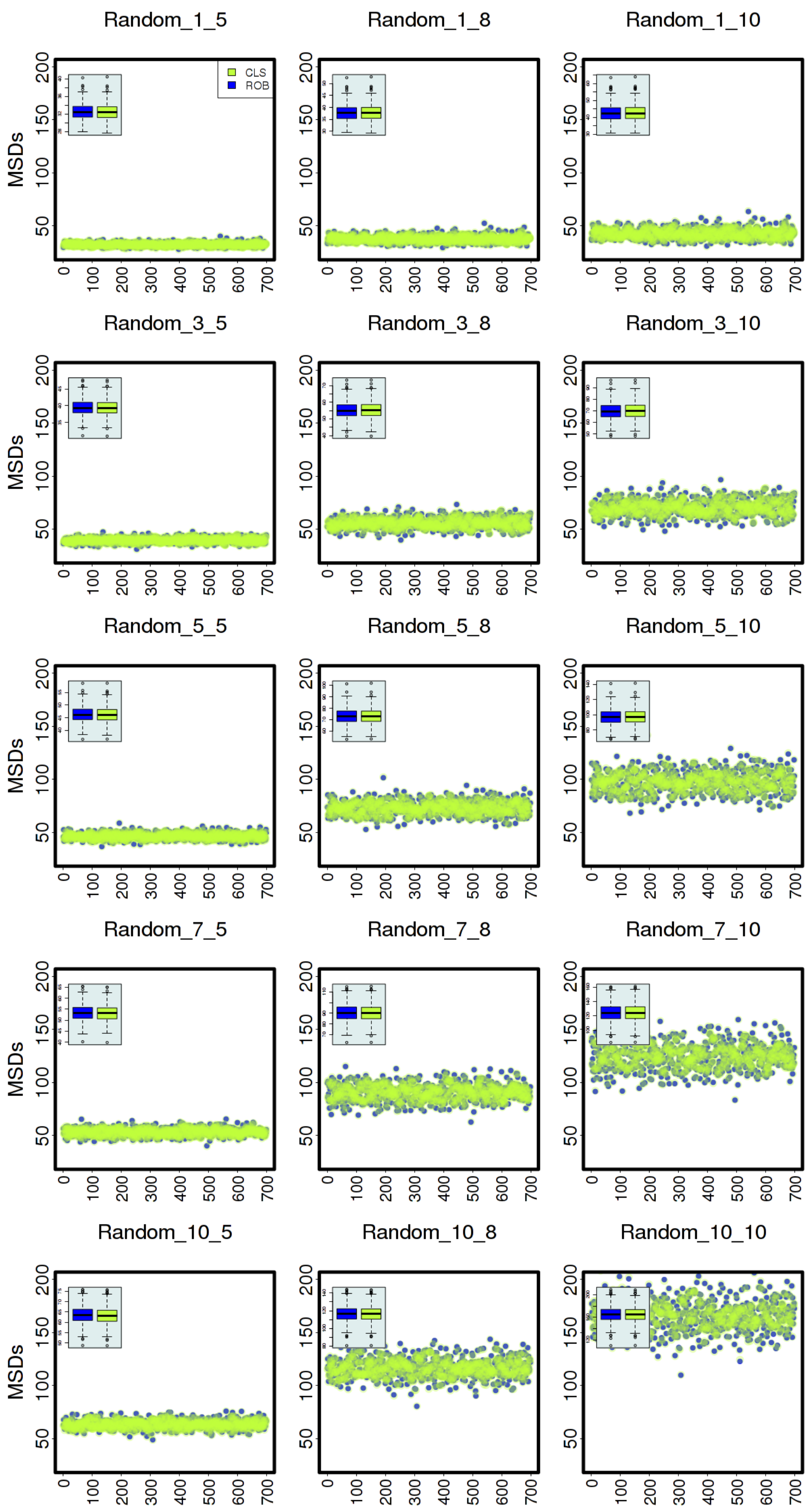

Figure S4. Plots of classical and robust $\mathrm{MSD}_{\mu}^{i} = \sum_{l=1}^{1000} \frac{(\widehat{\mu}_{il} - \mu_{il})^2}{1000}$ for each of the 698 genotypes for the **random** contamination scenarios (`1st stage`)

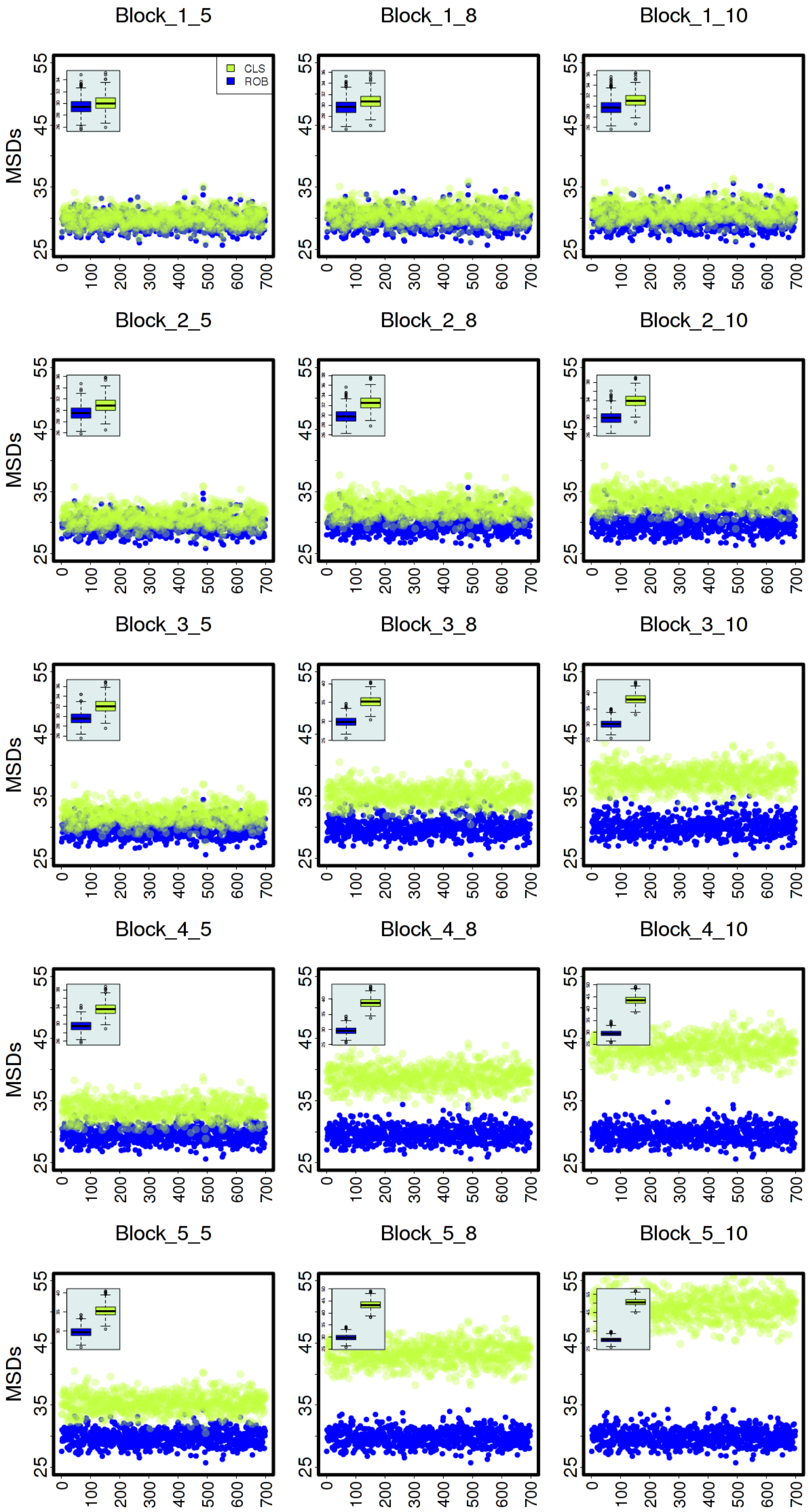

Figure S5. Plots of classical and robust $\text{MSD}_{\mu}^{i} = \sum_{l=1}^{1000} \frac{(\widehat{\mu}_{il} - \mu_{il})^2}{1000}$ for each of the 698 genotypes for the **block** contamination scenarios (`1st stage`)

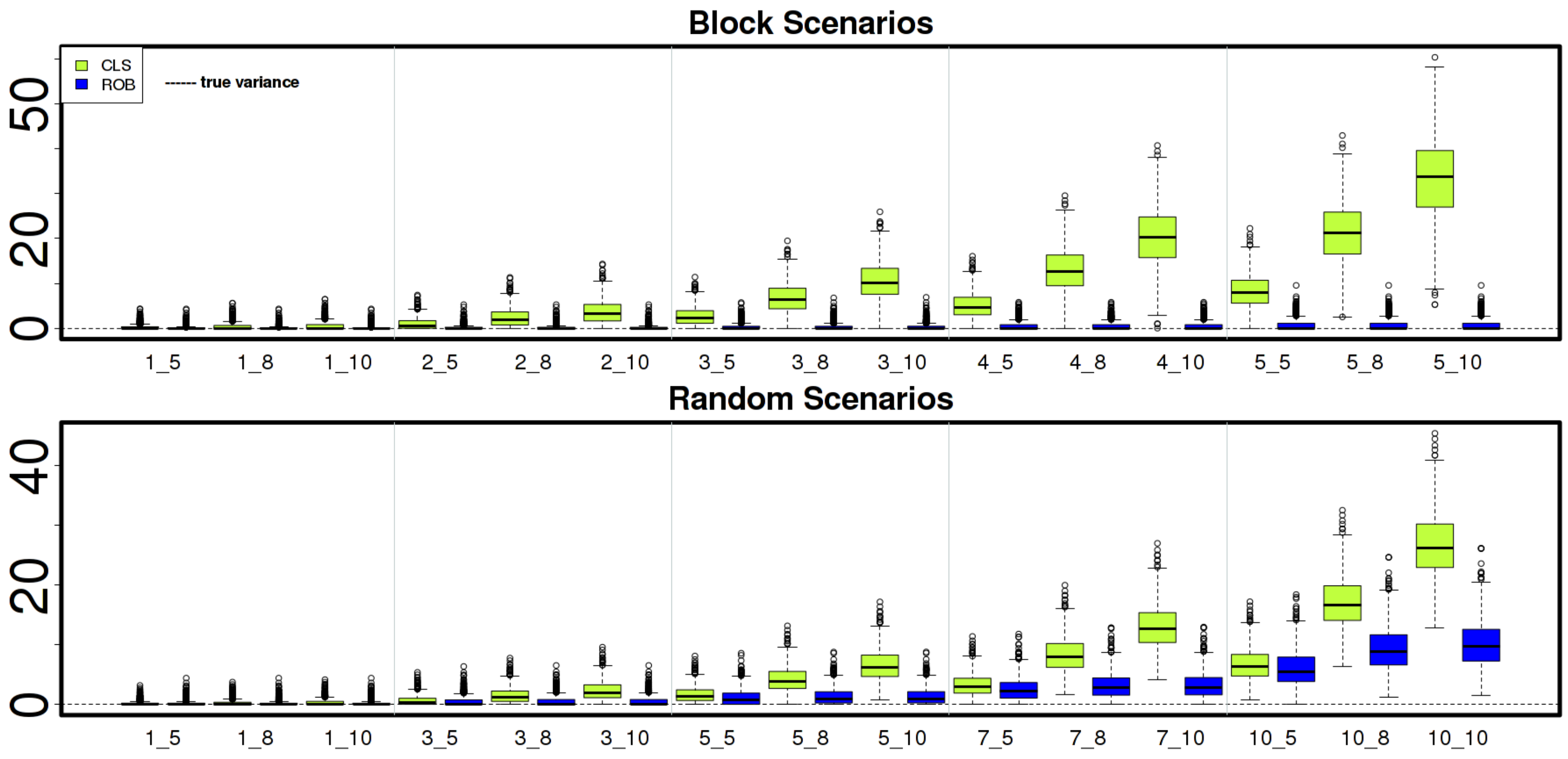

Figure S6. Boxplots of the 1000 classical and robust estimated variances for *replicates* (σ_r^2) for the **block** and **random** contamination scenarios (`1st stage`)

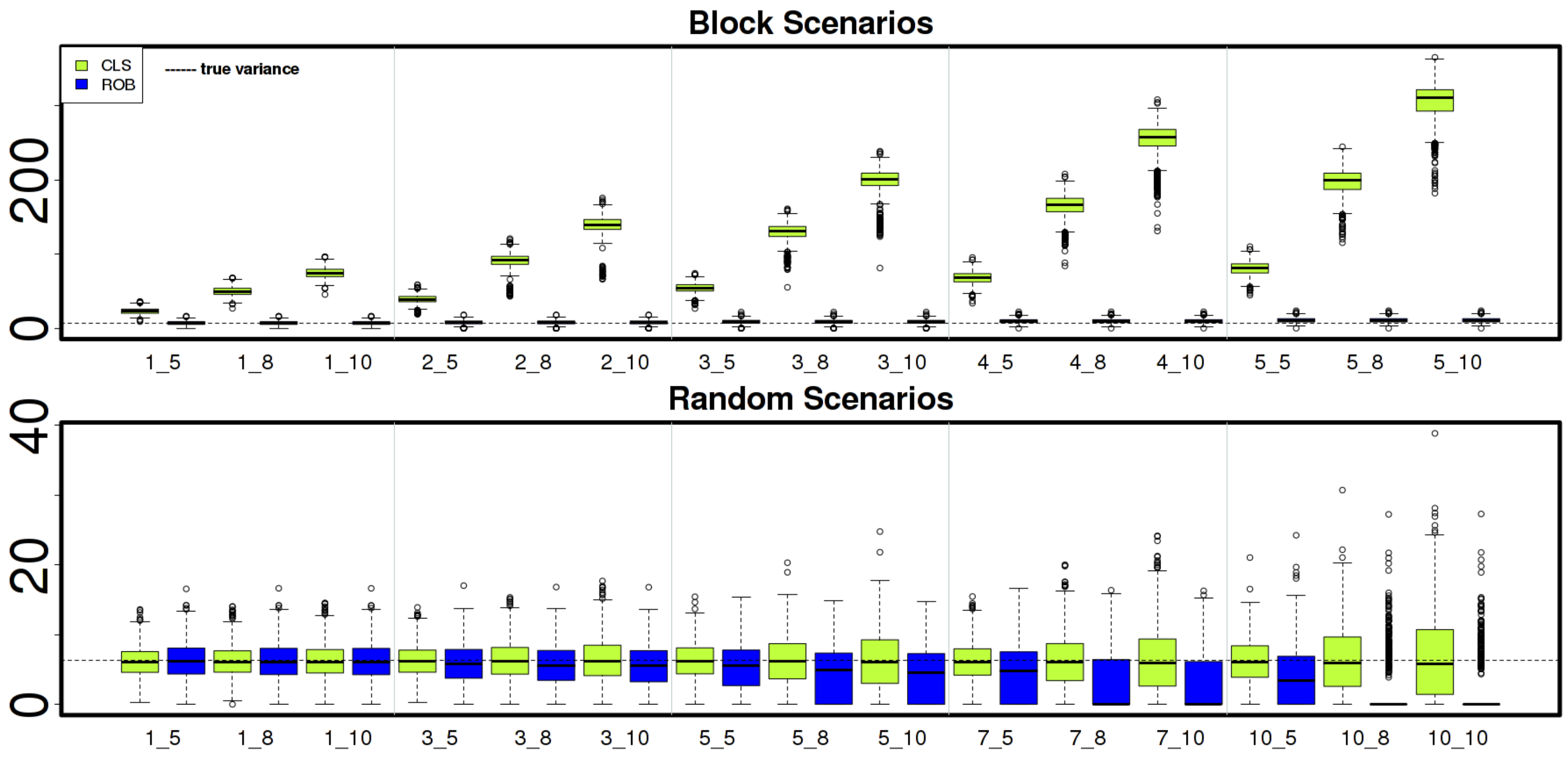

Figure S7: Boxplots of the 1000 classical and robust estimated variances for *block nested within replicates* ($\sigma^2_{r:b}$) for the **block** and **random** contamination scenarios (`1st stage`)

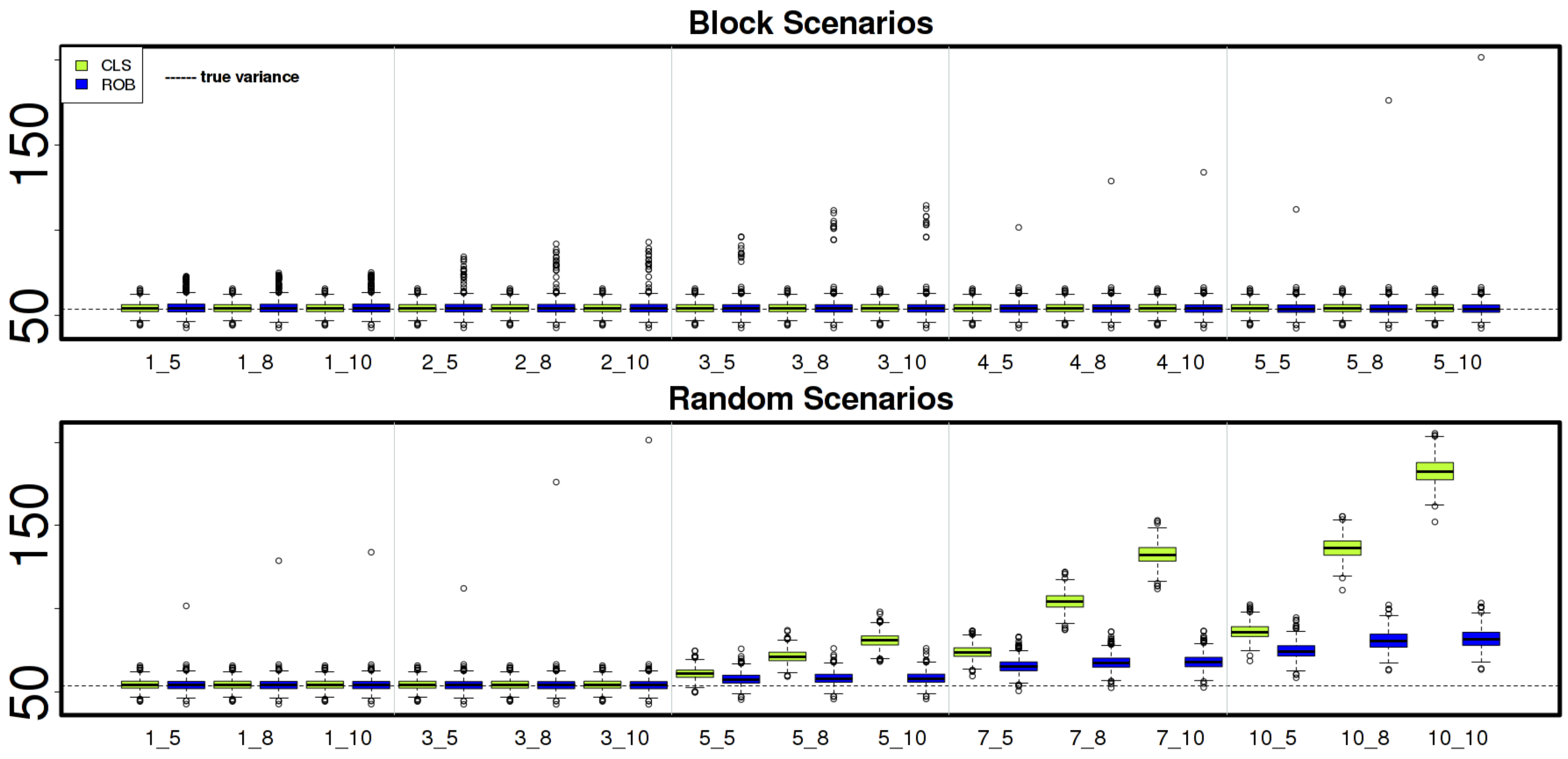

Figure S8: Boxplots of the 1000 classical and robust estimated variances for *residuals* (σ_e^2) for the **block** and **random** contamination scenarios (`1st stage`)

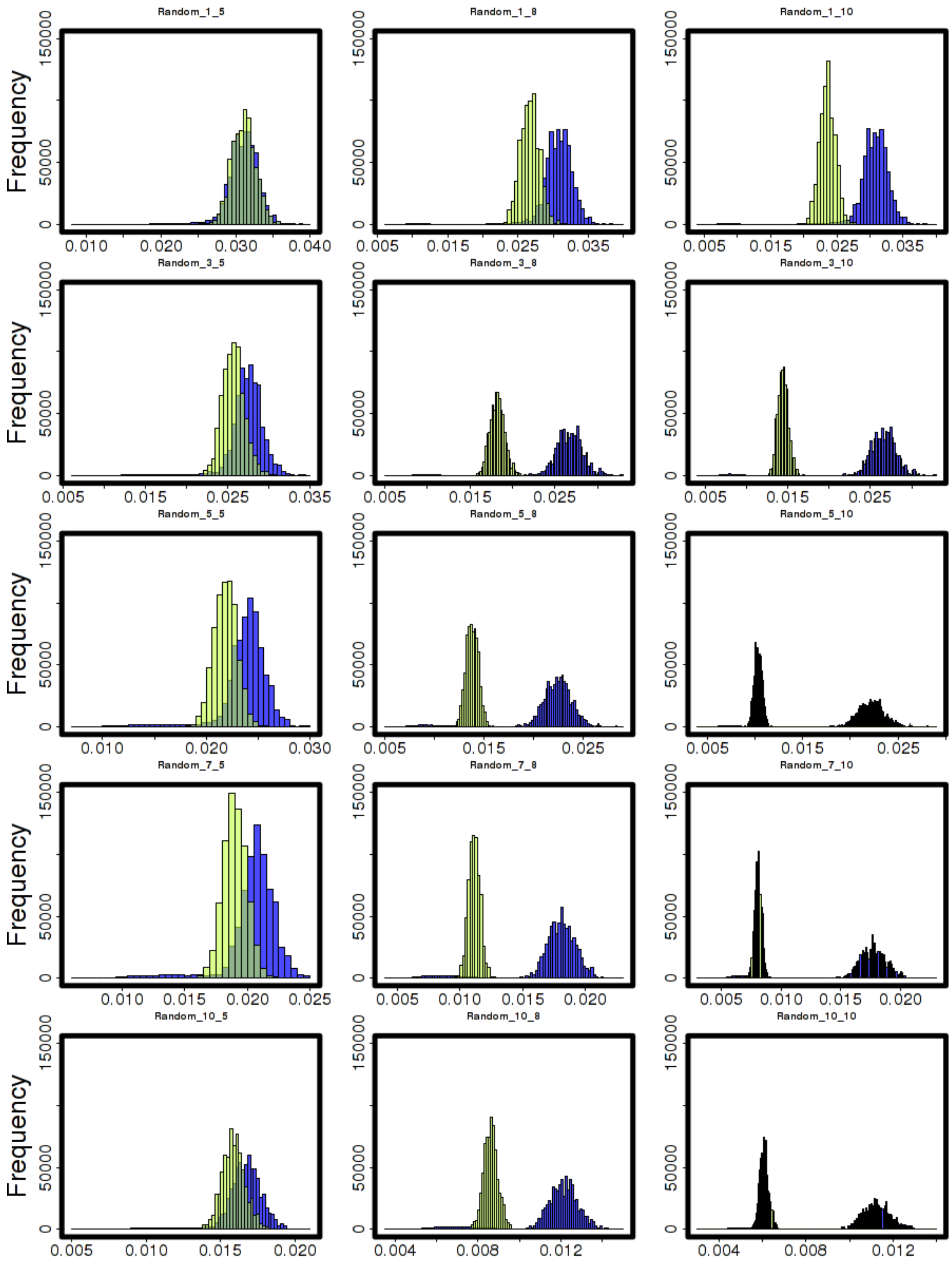

Figure S9. Histograms of the classical and robust Standard's weights for the **random** contamination scenarios (`1st stage`)

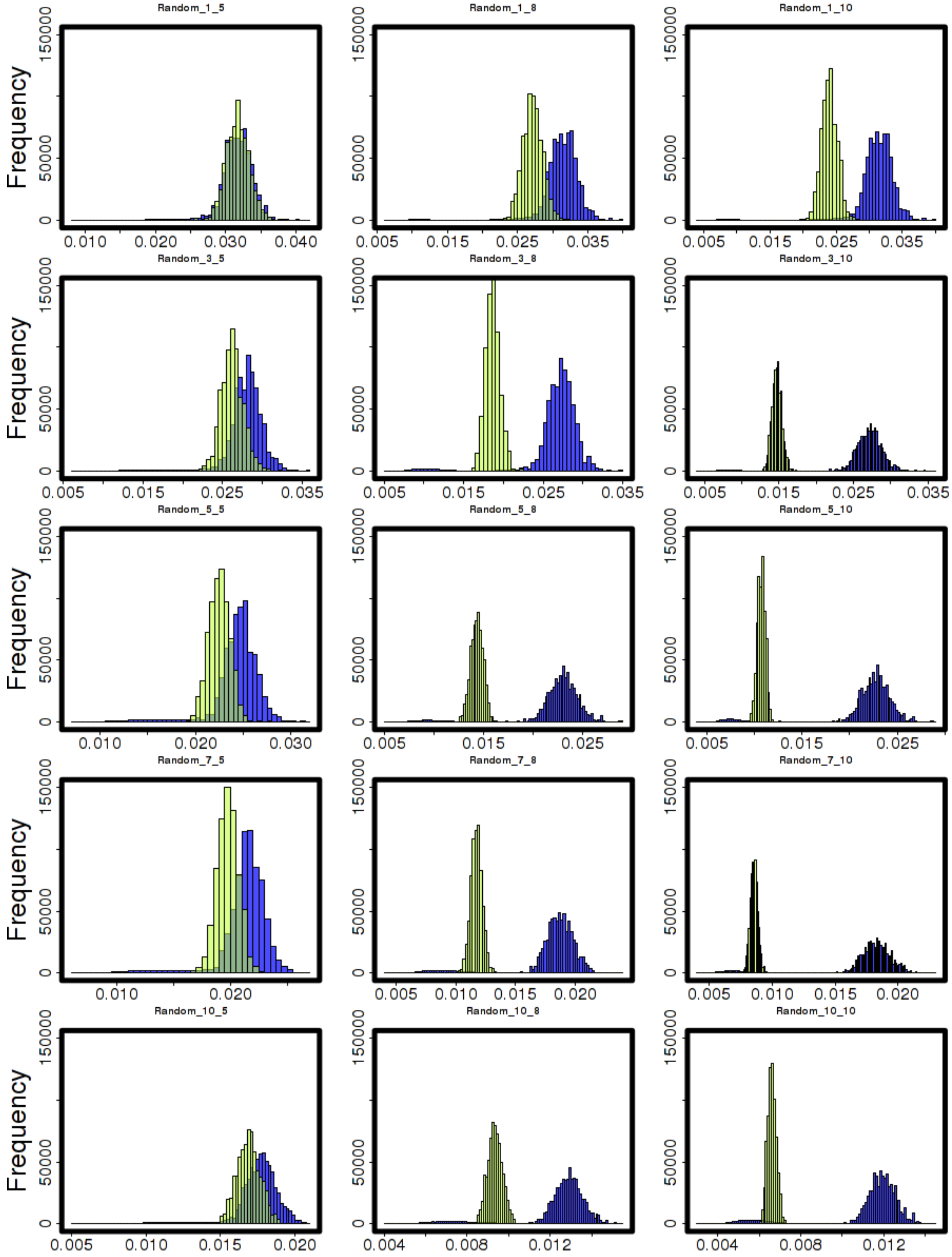

Figure S10. Histograms of the classical and robust Smith's weights for the **random** contamination scenarios (`1st stage`)

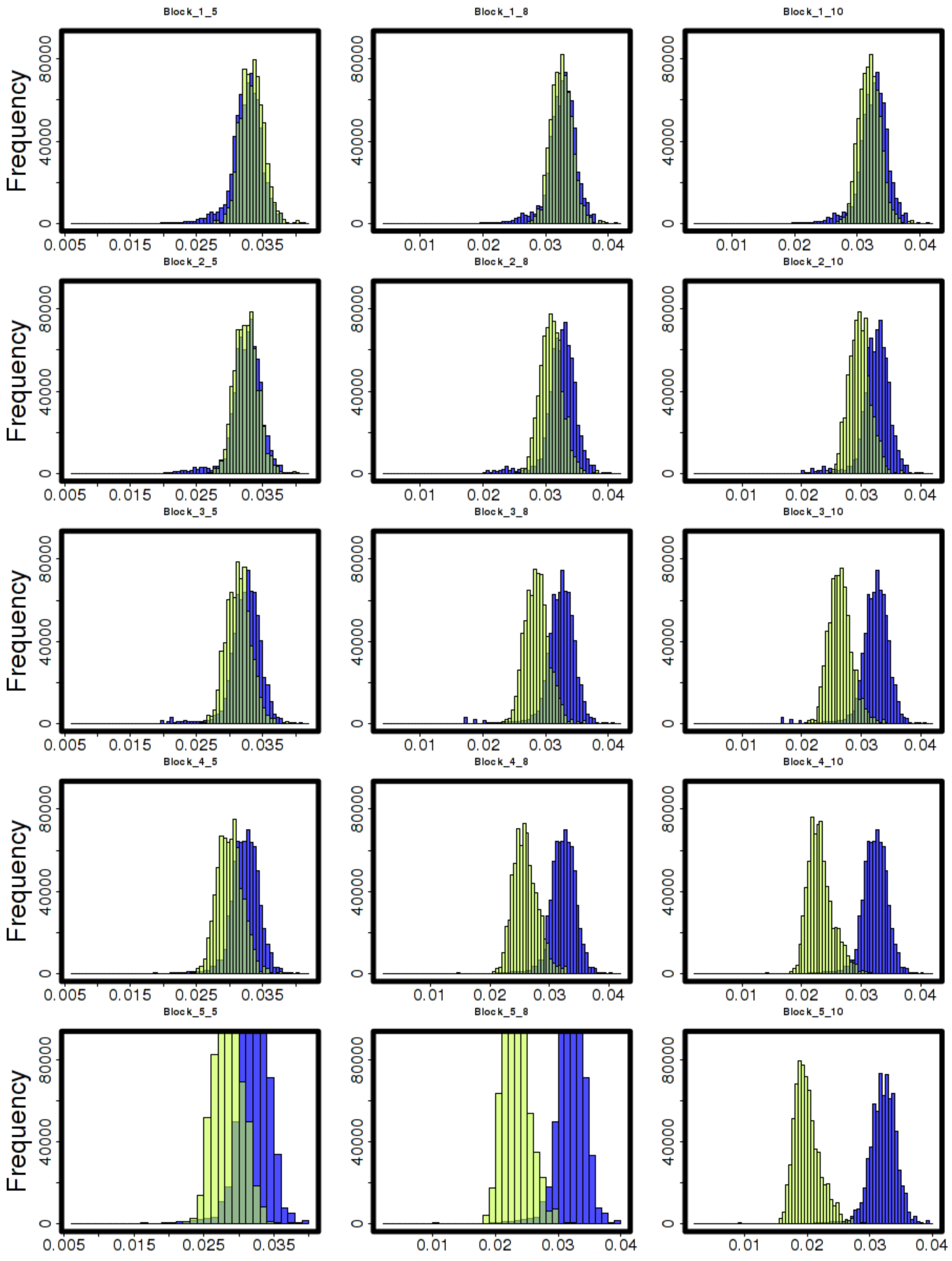

Figure S11. Histograms of the classical and robust Standard's weights for the **block** contamination scenarios (`1st stage`)

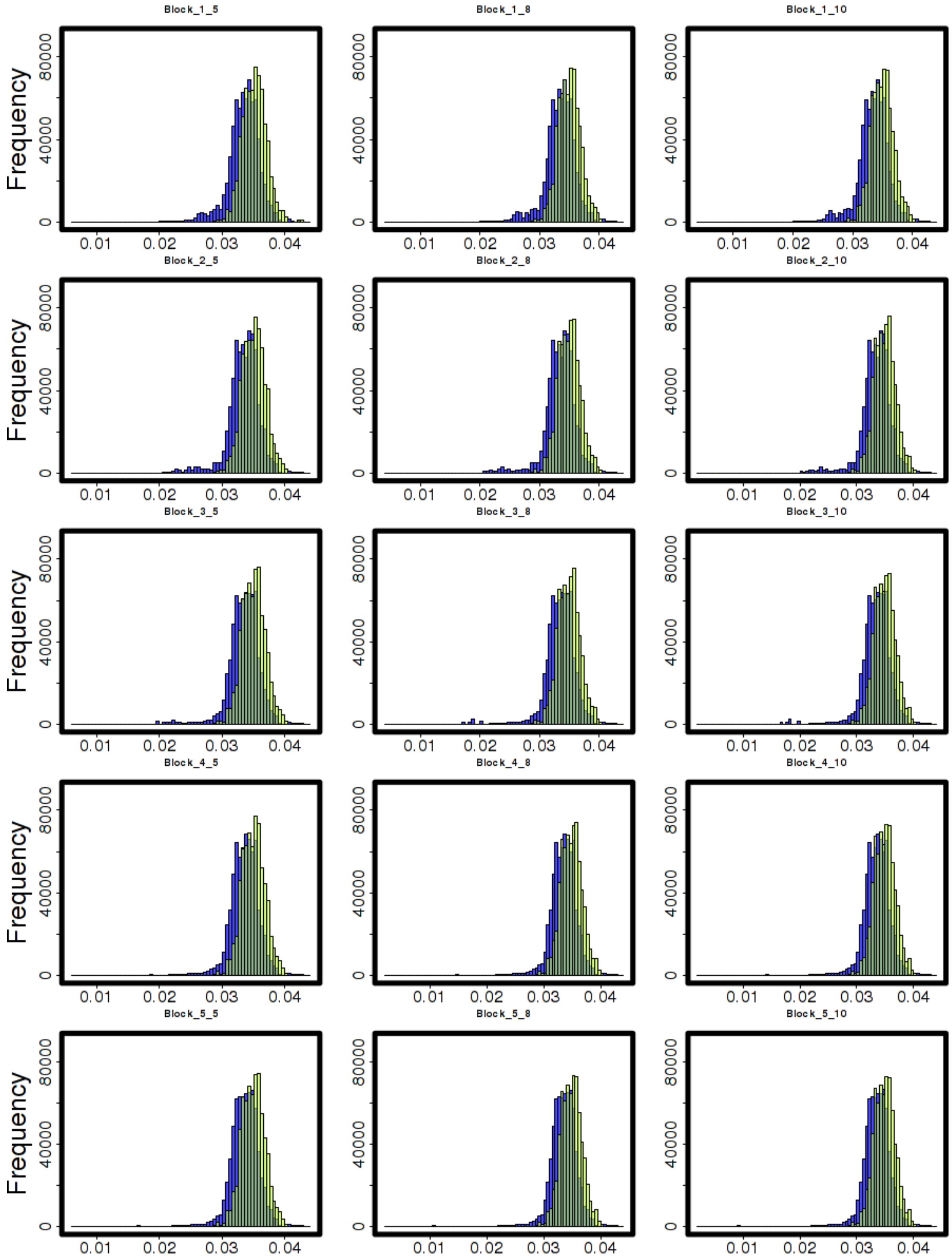

Figure S12. Histograms of the classical and robust Smith's weights for the **block** contamination scenarios (`1st stage`)

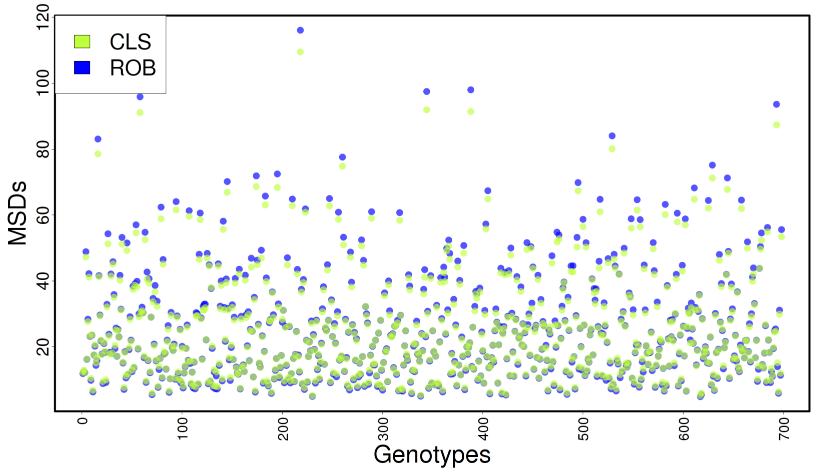

Figure S13. Plot of the classical and robust $\text{MSD}_g^i = \sum_{j=1}^{1000} \frac{(\widehat{g}_{ij} - g_{ij})^2}{1000}$ for each of the 698 genotypes for the **null** scenario (`2nd stage`)

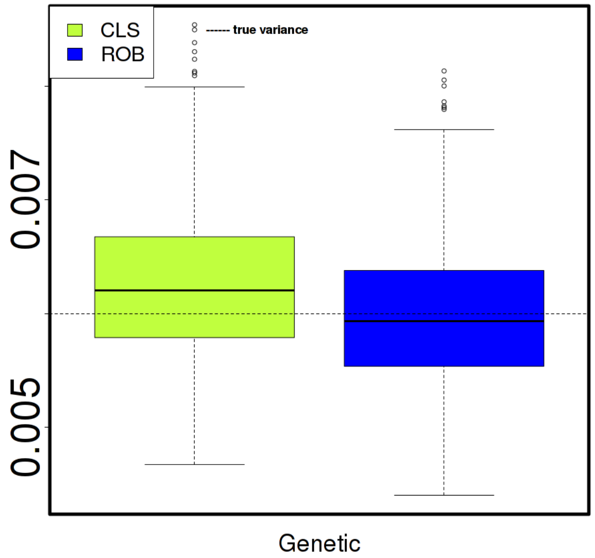

Figure S14. Boxplots of the 1000 classical and robust estimated *marker-effect* (σ_s^2) variances for the **null** scenario (`2nd stage`)

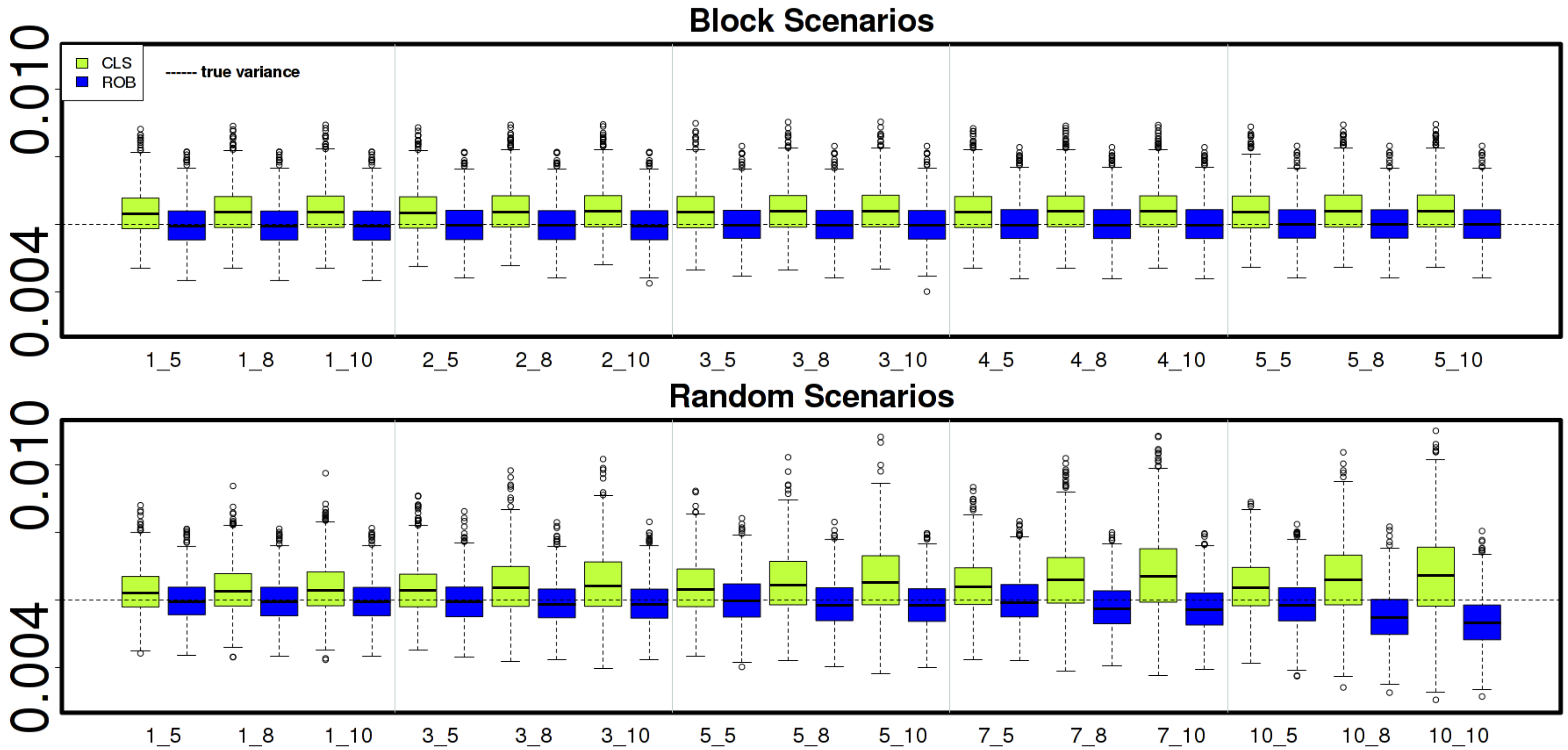

Figure S15: Boxplots of the 1000 classical and robust estimated *marker-effect* variances (σ_s^2) for the **block** and **random** contamination scenarios (`2nd stage`)

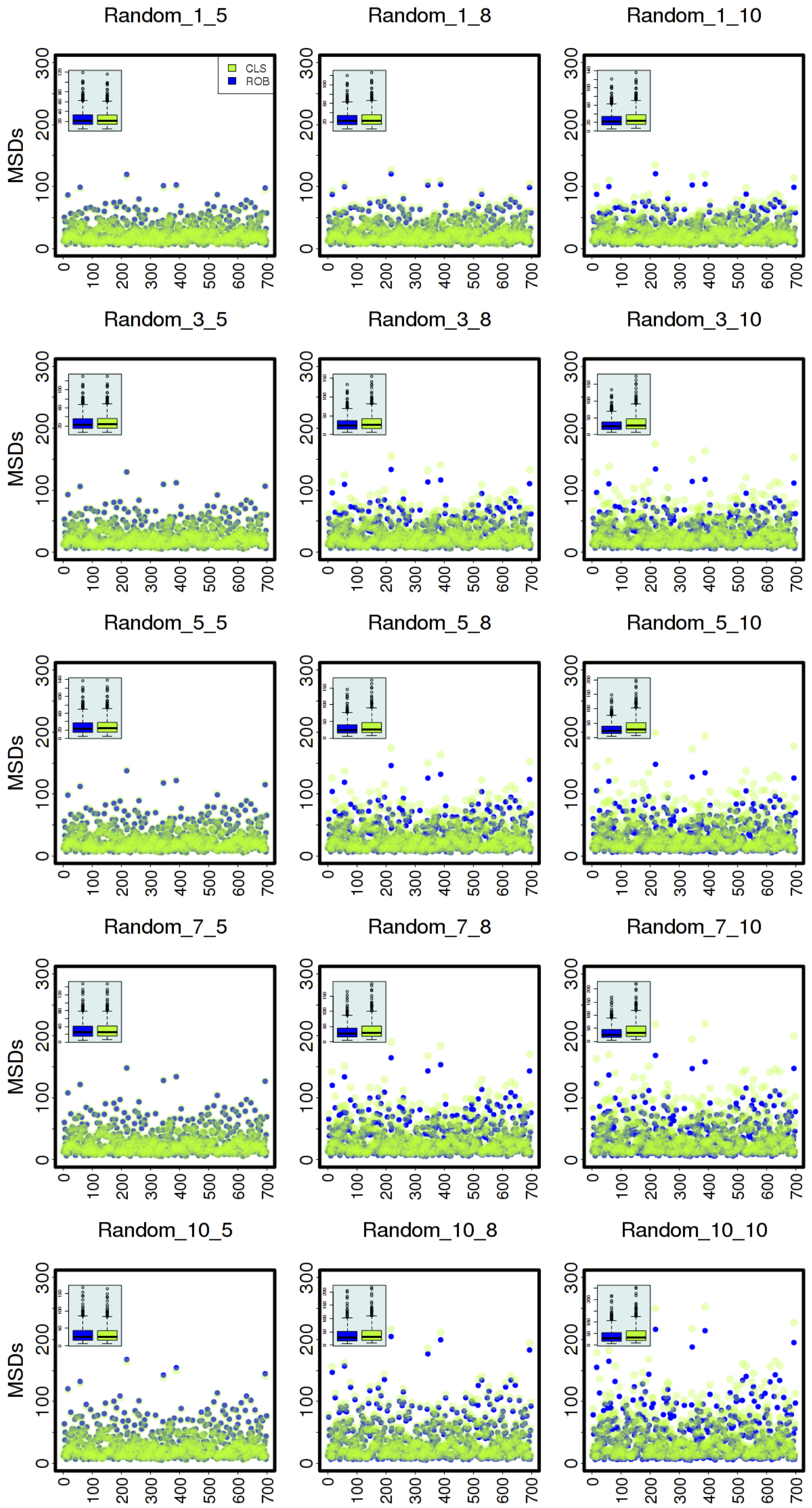

Figure S16. Plots of the classical and robust $\text{MSD}_g^i = \sum_{l=1}^{1000} \frac{(\widehat{g}_{il} - g_{il})^2}{1000}$ for each of the 698 genotypes for the **random** contamination scenarios (`2nd stage`)

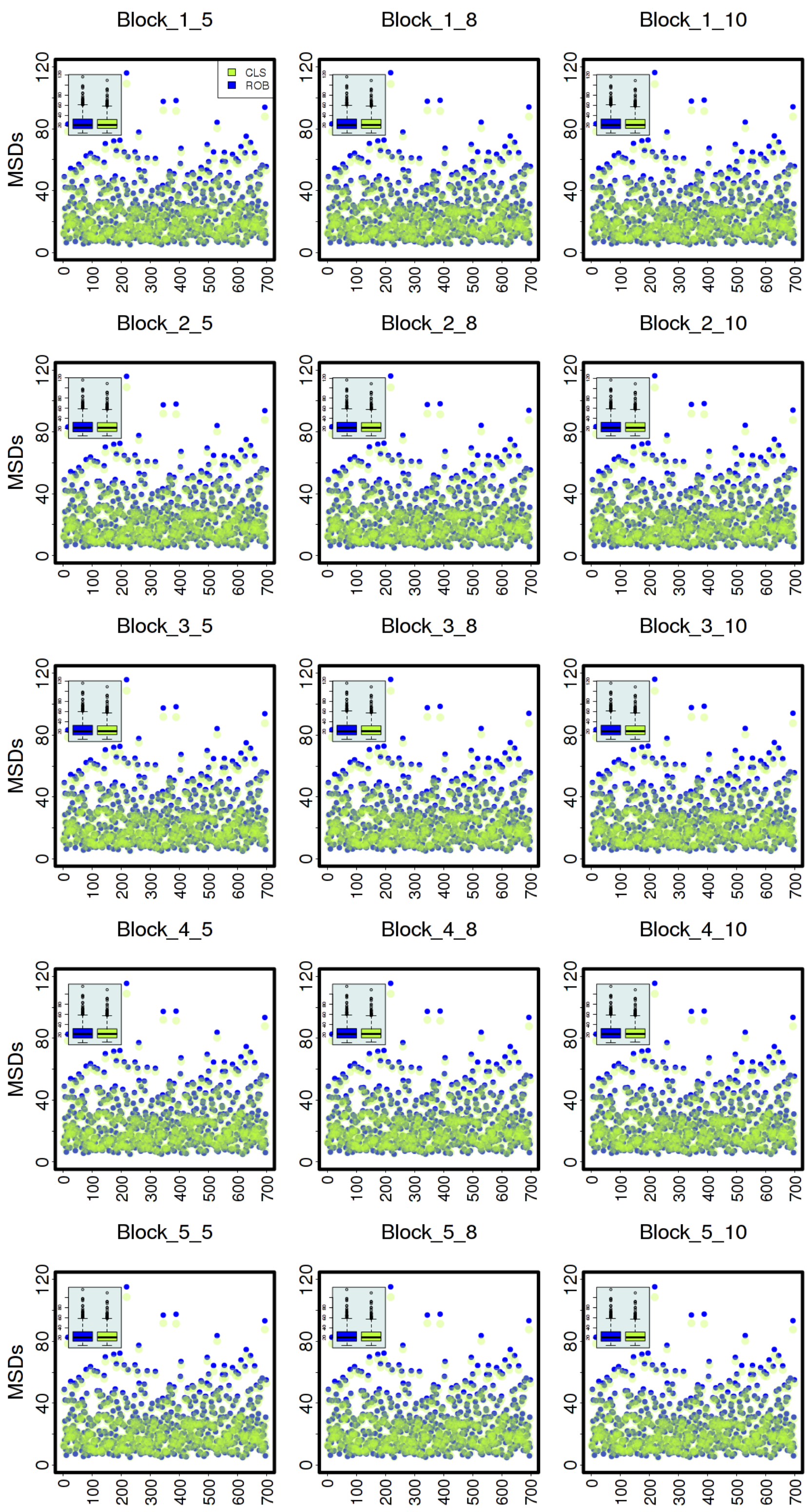

Figure S17. Plots of the classical and robust $\mathrm{MSD}_g^i = \sum_{l=1}^{1000} \frac{(\widehat{g}_{il} - g_{il})^2}{1000}$ for each of the 698 genotypes for the **block** contamination scenarios (`2nd stage`)

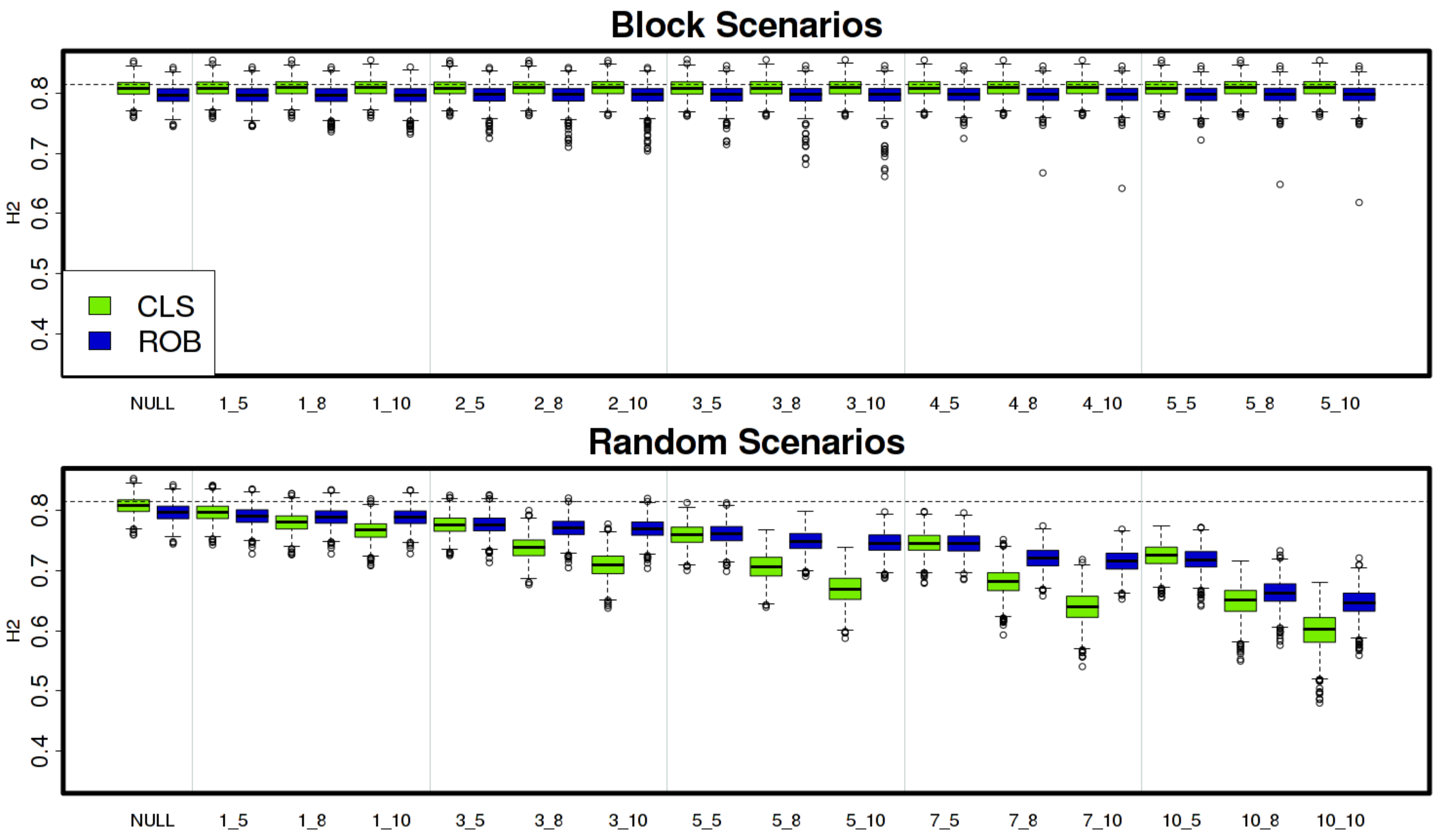

Figure S18. Boxplots of estimated heritabilities (H^2) computed by method **M5** for the **block** and **random** contamination scenarios (`3rd stage`)

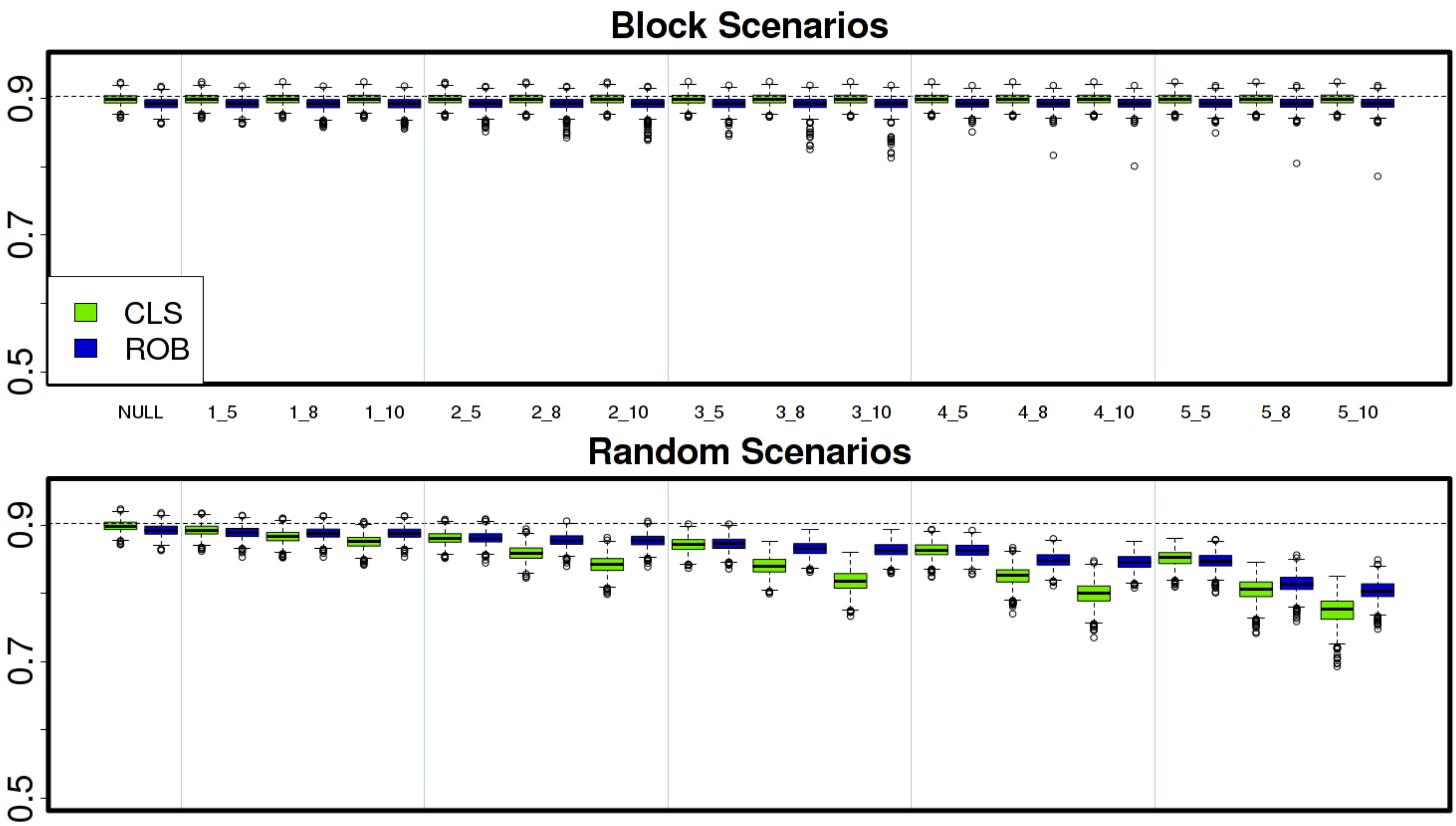

Figure S19. Boxplots of estimated predictive accuracies (PA) computed by method **M5** for the **block** and **random** contamination scenarios (`3rd stage`)

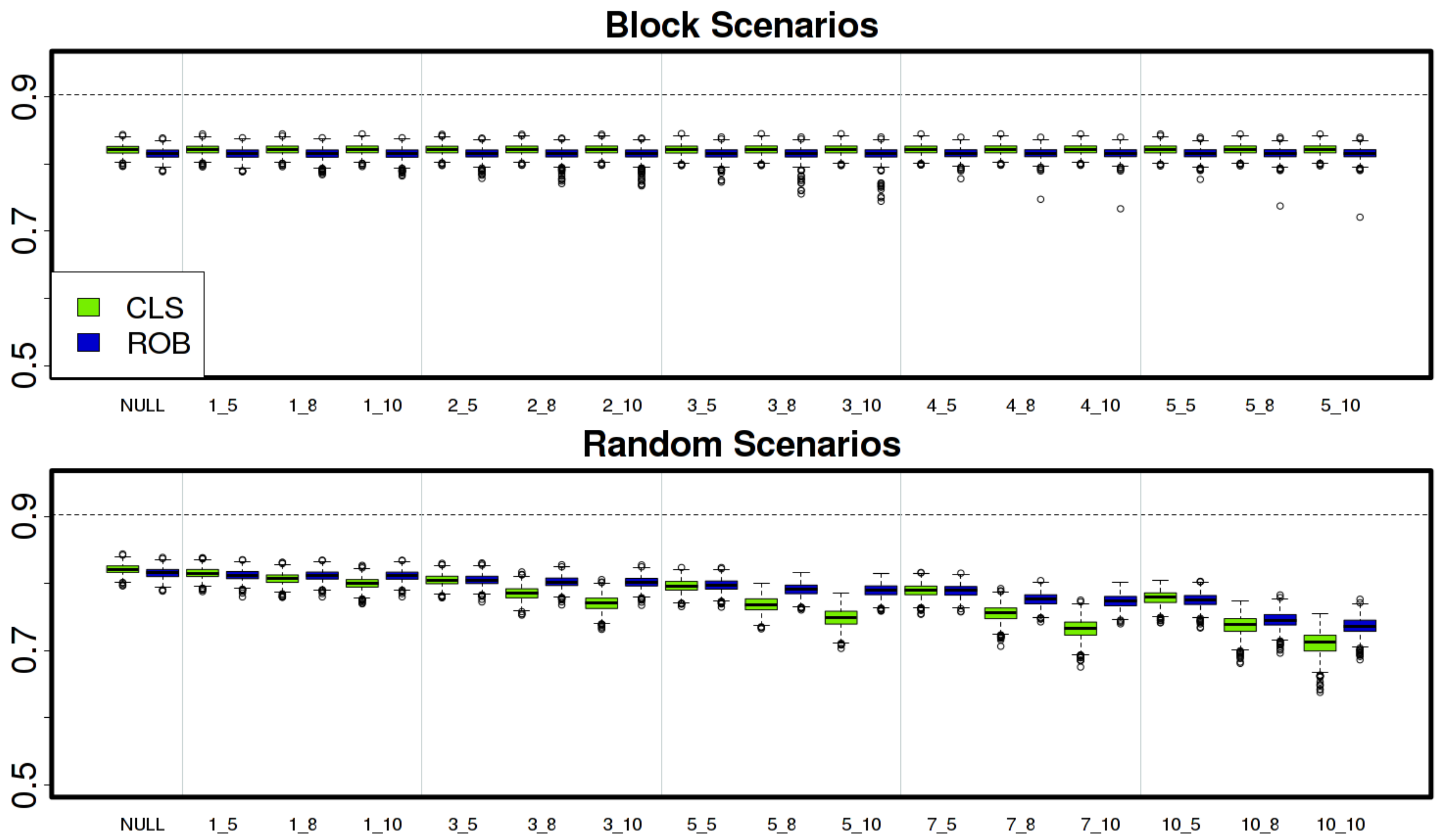

Figure S20. Boxplots of estimated predictive accuracies (PA) computed by method **M7** for the **block** and **random** contamination scenarios (`3rd stage`)
