# Supplementary material for: Robust estimation of heritability and predictive accuracy in plant breeding: evaluation using simulation and empirical data
Source: BMC Genomics. 2020 Jan 14;21:43. doi: 10.1186/s12864-019-6429-z (PMC6958597; doi:10.1186/s12864-019-6429-z)
Supplement: Supplementary file 3 — Additional file 3 This file contains a discussion of the differences between the second-stage results obtained using either the robust Smith’s weights or the doubly robust Smith’s weights. [file 12864_2019_6429_MOESM3_ESM.pdf]

Appendix C

Robust Smith's weights and doubly robust Smith's weights

The robust estimation procedure weights each observation according to its relative contribution to the model fit while fitting the model at the first stage. In this way it attempts to explain most of the variation in the data. Here, we use simulation to investigate whether using doubly robust Smith's weights accounts for outliers better than using robust Smith's weights. The simulation considers 698 genotypes each with two replicates for a total of 1396 observations. The simulation encompasses three scenarios, namely the *null* (0% contamination), *block* 3_8 (3.5% contamination) and *random* 7_8 (3.9% contamination) scenarios, and considers 250 simulation runs for each scenario. Only 250 simulation runs are considered for each scenario because the simulations are computationally very expensive. The *block* 3_8 and *random* 7_8 simulation scenarios were chosen because (i) they have approximately the same percentage of overall contamination and (ii) the 8-sd shift-outliers are considered sufficiently extreme in size. We expect the weights computed for each of the two replicate observations to differ substantially if only one of the pair is an outlier.

The Smith's weights are the diagonal elements of the inverse of the estimated variance-covariance matrix from the first-stage model. Because the robust fit approximates most rather than all the data, the variances of the outlying observations will be greater than those for the regular observations. Therefore, the key idea of doubly robust Smith's weights is to account for this effect by re-weighting the Smith's weights from the first stage, by using either the minimum (*min*) or mean (*mean*) of the robust Smith's weights assigned to each pair of replicate observations in the first stage. We denote the standard robust Smith's weights with SW, robust Smith's weights re-weighted by the minimum (SW_{min}) or the mean (SW_{mean}) of the weights assigned to the two replicate observations at the first stage.

Figure S1 C shows the differences in the distributions of the three different Smith's weights (SW, SW_{min} and SW_{mean}) computed across the 250 simulation runs for the *null*, *block* 3_8 and *random* 7_8 scenarios. Notably, the weights for the *random* scenario are clearly smaller than those for either the null or block scenarios.

The mean squared deviations of SW_{min} from the SW_{mean} across the three scenarios and the 250 simulation runs is approximately 0.4×10^{-11} for the *null* scenario, 0.2×10^{-10} for the *block* 3_8 scenario and 0.7×10^{-10} for the *random* 7_8 scenario. Figure S2 C (first row) further corroborates the observation that the SW_{min} and SW_{mean} produce virtually the same results. However, there are noticeable differences between either of the re-weighted robust Smith's weights and the robust Smith's weights across all the three scenarios (Figure 2C, second-row). Overall, the robust Smith's weights are generally higher than the re-weighted ones. Furthermore, despite the MSDs between the robust Smith's weights and the re-weighted Smiths weights being small, they are 10^5 to 5×10^5 times greater than the MSDs of the Smith's weights re-weighted with *min* from those for the Smith's weights re-weighted with *mean*.

Figure S3 C, shows that for the 250 simulation runs for the *null*, *block* 3_8 and *random* 7_8

scenarios, there are no major differences between the estimated genetic variances obtained at the second-stage by using either the *min* or *mean* of the weights assigned to the two replicate observations to re-weight the robust Smith's weights from the first-stage model fit. In addition, the re-weighting scheme is most effective for the *random* contamination scenario for which using the robust Smith's weights alone is not sufficient to produce unbiased estimates of the true genetic variance.

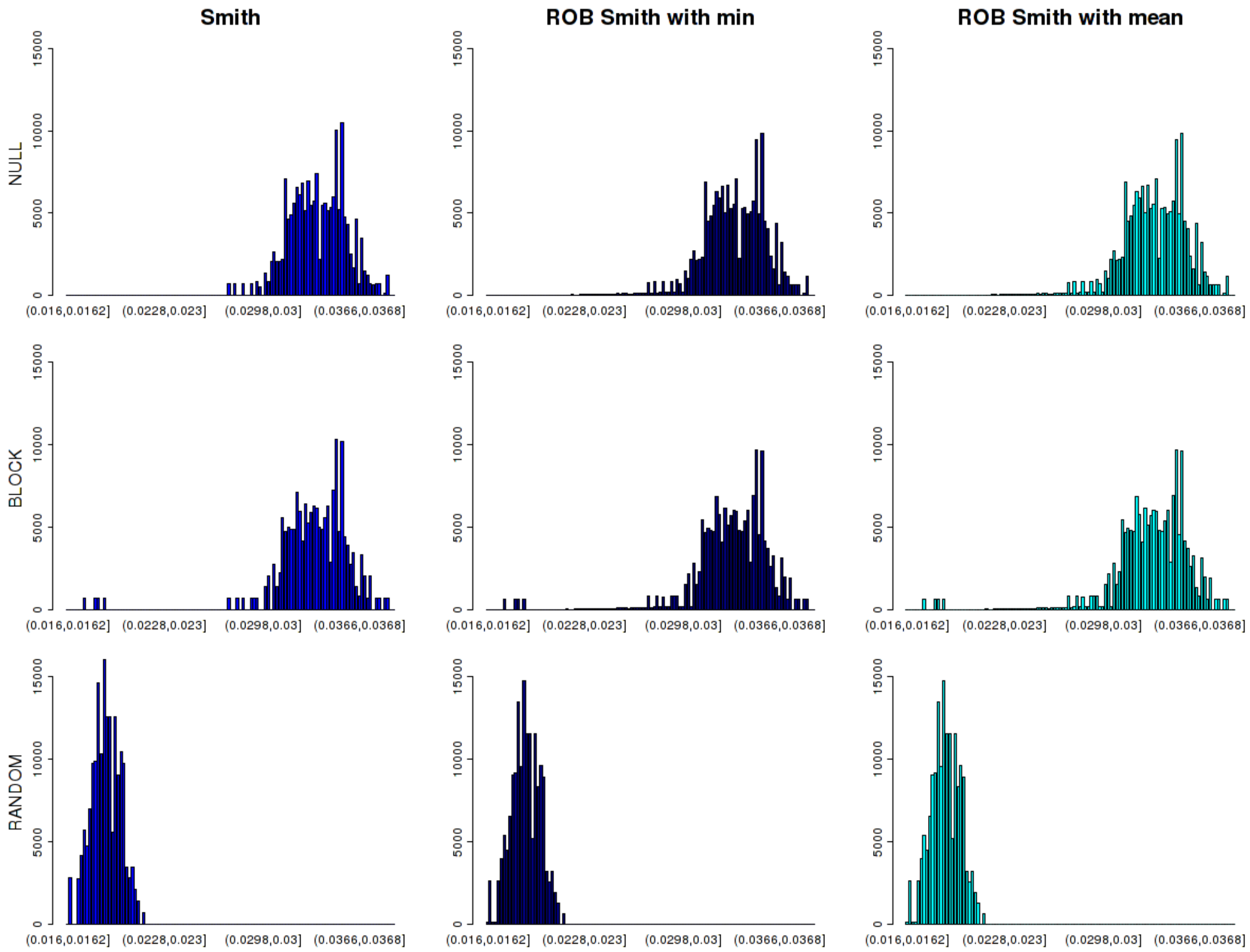

Figure S1 C: Frequency histograms of the Smith's weights computed from the first-stage robust model fit with and without re-weighting using the robust weights, for the *null*, *block* 3_8 and *random* 7_8 scenarios. The same number of bins and binwidths were used to facilitate comparison.

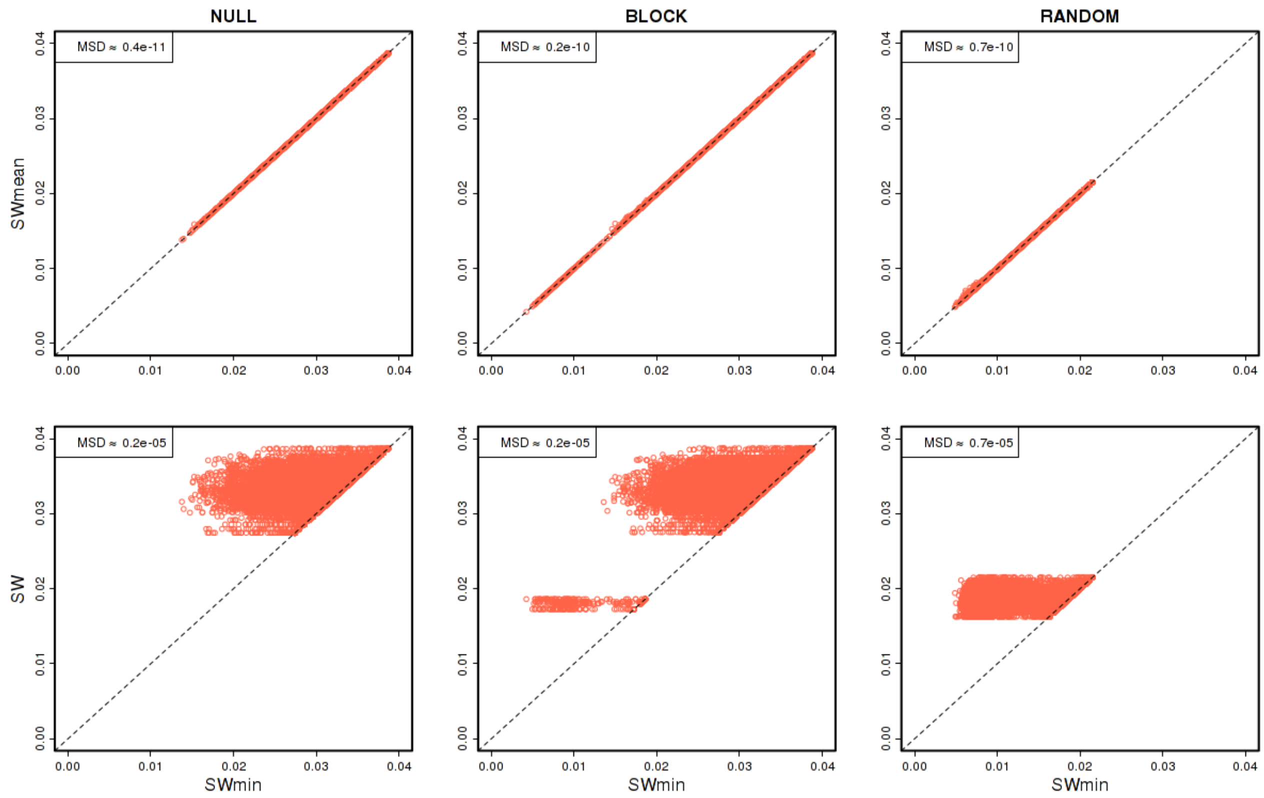

Figure S2 C: Plots of the Smith's weights computed from the first-stage robust fit with and without the re-weighting using the robust weights, for the *null*, *block* 3_8 and *random* 7_8 scenarios. Re-weighted Smith's weights with *min* (SW_{min}) are plotted against the re-weighted Smith's weights with *mean* (SW_{mean}; first row) and re-weighted Smith's weights with *min* (SW_{min}) are plotted against the Smith's weights (SW).

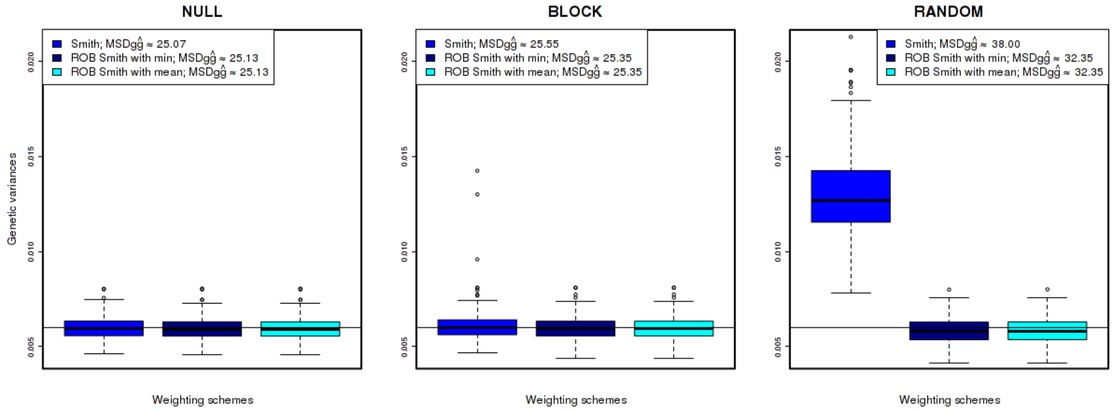

Figure S3 C: Boxplots of the 250 estimated robust genetic variances using the ordinary Smith's weights (as computed from the robust first-stage model; robust Smith's weights) and the weighted Smith's weights (computed by reweighting the Smith's weights with the robust weights otained from the robust fit using both the minimum and the mean of the weights across the two replicates; doubly robust Smith's weights) for the *null*, *block* 3_8 and *random* 7_8 scenarios; Mean squared deviations (MSDs) from the estimated breeding values (EBVs) to the true breeding values (TBVs) are also shown.
