# Supplementary material for: Robust estimation of heritability and predictive accuracy in plant breeding: evaluation using simulation and empirical data
Source: BMC Genomics. 2020 Jan 14;21:43. doi: 10.1186/s12864-019-6429-z (PMC6958597; doi:10.1186/s12864-019-6429-z)
Supplement: Supplementary file 4 — Additional file 4 This file contains the R code needed to generate phenotypic data as well as SNP marker data and kinship, computed from an alpha-design as the one of the maize dataset used in the simulations. [file 12864_2019_6429_MOESM4_ESM.pdf]

Appendix D

R code for generating the synthetic dataset, i.e., the toydata

```
## The R code below allows the user to generate phenotypic data from an alpha-design
## as well as generate SNP marker data and kinship matrix from it;
## This is because we cannot share the true SNP data and kinship matrix

set.seed(9999999)
library(agricolae) # for using the design.alpha() function; needs the right R version installed
library(dplyr)     # enables the use of operator %>%

## generating an alpha-design with 702 genotypes and 39 blocks each with size 18
## NOTE that in our case, the simulated datasets had 698 genotypes
## in which case positions 699--702 in the design were set to missing values
  # defining the parameters for the design
    trt <- 1:702  # number of treatments, i.e., genotypes
    k   <- 18     # block size
    r   <- 2      # nubmer of replicates
    # s <- trt[702]/k    # number of blocks; don't need to specify this

  # creating the design, which is only ~ 90% efficient
  # the user can create more efficient alpha-desgins by using the program CycDesignN
    outdesign <-  design.alpha(trt, k, r, serie = 2, seed = 0, kinds = "Super-Duper",
                          randomization=TRUE)

  # getting the dataset obtained from the design
    ourdata<-outdesign$book[,c(2,5,3,4)]
    # changing colnames
      colnames(ourdata)<-c("plot","rep","block","geno")
    # changing blocks 40:78 to 1:39 (this is actually not needed)
      ourdata[703:1404,3]<-as.integer(ourdata[703:1404,3])-39

## simulating maize yield
  # fixing the variances for the synthetic data
    var_s <- 0.005892 # marker effect variance
    var_b <- 6.3148   # block effect variance
    var_e <- 53.8715  # residual variance

  # simulationg the SNP effects from N(0,var_s)
    set.seed(79753)
    u<-rnorm(11646,0,sqrt(var_s))

  # generating SNP marker 702x111646 matrix (in this case a matrix of 1s and -1s)
  # as well as the kisnhip matrix
  # generating the SNP markers as below is just an had-doc way of doing it;
  # it assumes the SNPs are independent and does not account for population structure
    Z<-matrix(rbinom(n=702*11646,size=1,prob=0.5),702,11646)
    for(i in 1:702){for(j in 1:11646){Z[i,j]<-2*Z[i,j]-1}}
    K<-Z%*%t(Z) # or K<-Z%*%t(Z)/11646

  # computing the true 702 breeding values
    g<-Z%*%u
```

```
# computing plot and block effects for the 2 replicates
  # block effect is the same within each of the 39*2 blocks
  # but different across all the 39*2 blocks
  block_eff <- vector()
   for(i in 1:78){block_eff<-c(block_eff,rep(rnorm(1,0,sqrt(var_b)),18))}
  res_eff   <- rnorm(702*2,0,sqrt(var_e))
  plot_eff  <- block_eff+res_eff
  ourdata   <- cbind(ourdata,block_eff,res_eff,plot_eff)

# computing yield values for the 2 replicates; first 702 pos are rep1; last 702 are rep 2
# the TBVs are the same for the 702 genotypes on both replicates
  dataset   <- (ourdata[order(ourdata$rep,ourdata$geno),])
  dataset   <- cbind(dataset,rep(g,2))
    colnames(dataset)[8]<-c("TBVs")
  yield     <- dataset[,7]+dataset[,8]
  dataset   <- cbind(dataset,yield)

  # # UNCOMMENT THE CODE BELOW IF YOU WANT TO CONTAMINATE THE DATA
  #   # RANDOM contamination of a % of observations on replicate 1
  #     # define the % of contamination, e.g.,
  #       perc.cont<-0.05
  #     # compute the number of outliers referring to that %
  #       n.outliers<-round(702*perc.cont)
  #     # randomly chose the positions/genotypes to contaminate
  #     # in this case, because we ordered the dataset, the positions to contaminate
  #     # match the genotypes of the first replicate with the same index
  #       outlier.positions<- sample(1:702,n.outliers)
  #     # replace the observations in the assigned outlier.positions
  #     # by their value plus, e.g., 5 times the standard deviation
  #       dataset[outlier.positions,9]<-dataset[outlier.positions,9]+5*sqrt(var_e)
  #   # whole BLOCK contamination on replicate 1
  #     # define the number of blocks to contaminate, e.g.,
  #       n.blocks<-3
  #     # randomly chose the blocks on the first replicate that should be contaminated
  #     # the number of contaminated observations = n.blocks*18
  #       outlier.blocks<- sort(sample(1:39,n.blocks))
  #     # replace the observations in the assigned outlier.blocks
  #     # by their value plus, e.g., 5 times the standard deviation
  #       for(i in 1:n.blocks){
  #         dataset[dataset$block==outlier.blocks[i]&dataset$rep==1,9]<-
  #           dataset[dataset$block==outlier.blocks[i]&dataset$rep==1,9]+5*sqrt(var_e)
  #       }

# sorting the dataset by geno so that the robust 2-stage R code can be applied directly
  toydata<-dataset[order(dataset$geno),]
  toyG<-K
# saving the dataset
  save(toydata,toyG,file="toydata.Rdata")
```
