# Supplementary material for: Robust estimation of heritability and predictive accuracy in plant breeding: evaluation using simulation and empirical data
Source: BMC Genomics. 2020 Jan 14;21:43. doi: 10.1186/s12864-019-6429-z (PMC6958597; doi:10.1186/s12864-019-6429-z)
Supplement: Supplementary file 5 — Additional file 5 This file contains the R code needed to run both the classical and robust two-stage approaches. [file 12864_2019_6429_MOESM5_ESM.pdf]

Appendix E

R code for the classical & robust two-stage approaches

```
## NOTE:
## before running the code below one must first run the code that generates the toydata
## which is in the supplementary AppendixD_Rcode.pdf file

## loading the data
  load("toydata.Rdata")

## loading the libraries
  library(lme4)         # for classical LMM
  library(robustlmm)    # for robust LMM
  library(psych)        # to being able of using the tr() function
  library(asreml)       # for the classical LMM with kinship matrix
  # if tr() does not work load library(matrixcalc) and use matrix.trace() function

## CLASSICAL first-stage fit below is commented;
## uncomment when you wish to run it, in which case you should comment the robust fit

# ## fitting the first-stage classical model
#   fit<-  lmer(yield ~ -1 + geno + (1|rep)+(1|rep:block), toydata)
#     # getting the lsmeans and var-covar structure
#       R  <- summary(fit)$vcov
#       mu <- summary(fit)$coefficients[,1]
#     # computing the Smith's and Standard weights
#       w     <-(1/diag(R))      #Standard weights
#       wsmith<-diag(solve(R))   #Smith's weights
#       rm(R)
#     # keeping also the estimated random effects variances
#       STDs<-matrix(0,3,1)
#       STDs[1,1]<- attr(VarCorr(fit)$'rep:block', "stddev")
#       STDs[2,1]<- attr(VarCorr(fit)$'rep', "stddev")
#       STDs[3,1]<- attr(VarCorr(fit),"sc")
#       colnames(STDs)<-"std"
#       rownames(STDs)<-c("REP:BLOCK","REP","Residual")
#       stage1.vars<-STDs^2
#       rm(STDs)

## fitting the first-stage robust model
  fit<-  rlmer(yield ~ -1 + geno + (1|rep)+(1|rep:block), toydata,
             rho.sigma.e = psi2propII(smoothPsi, k = 2.28))
    # getting the lsmeans and var-covar structure
      R  <- summary(fit)$vcov
      mu <- summary(fit)$coefficients[,1]
    # getting the robust weights
    # do not confuse these with the Smith's and Standard weights
    # Note that if your data has missing values of yield, no robust weights are estimated
    # and therefore the process of getting the robust weights for the 2nd-stage
    # will not be as straighforward as it is in this case
      rob.weights<-getME(fit,name="w_e")
    # the robust weights need not the same for genos in rep1 and rep2
    # but we want only 1 robust weight per-genotype
```

```
    # thus we will choose the min between the 2 robust weights from the 2 replicates
    # the next computations need to be adpated for each dataset because the order of
    # the weights matches the one of the dataset as also do the order of the residuals
      n<-length(mu)
      aux<-vector()
      for(k in seq(1,(n*2-1),by=2)){aux<-c(aux,min(rob.weights[k],rob.weights[k+1]))}
      rob.weights<-aux
        rm(aux,k,n)
    # computing the Smith's and Standard weights, which incorporate the robust weights
      w      <-(1/diag(R))*rob.weights       #Standard weights
      wsmith <-diag(solve(R))*rob.weights    #Smith's weights
        rm(rob.weights,R)
    # keeping also the estimated random effects variances
      STDs<-matrix(0,3,1)
      STDs[1,1]<- attr(VarCorr(fit)$'rep:block', "stddev")
      STDs[2,1]<- attr(VarCorr(fit)$'rep', "stddev")
      STDs[3,1]<- attr(VarCorr(fit),"sc")
      colnames(STDs)<-"std"
      rownames(STDs)<-c("REP:BLOCK","REP","Residual")
      stage1.vars<-STDs^2
        rm(STDs)

## fitting the second-stage model -- classical approach used
  # # try out G=I to see how H2.M5 and H2.Oakey match
  # toyG<-diag(dim(toyG)[1])
  # colnames(toyG)<-names(mu)
  # rownames(toyG)<-names(mu)

  # preparing the data
    plantid<-names(mu)
    colnames(toyG)<-names(mu)
    rownames(toyG)<-names(mu)
    inv.toyG<-solve(toyG)
    ourdata<-data.frame(plantid = plantid,
                        mu = mu,
                        wsmith = wsmith,
                        w = w)
  # fitting the model
  # one can change the Smith's weights (wsmith) for the Standard weights (w) below
    fit.cls <-asreml( data    =ourdata,
                      fixed   =mu ~ 1,
                      random  =~ giv(plantid) ,
                      rcov    =~ units, na.method.Y = "include",
                      weights = wsmith,
                      family  = asreml.gaussian(dispersion=1.0),
                      control =asreml.control(workspace=16e7, ginverse=list(plantid=inv.toyG),
                                        maxiter=1000)
                    )
    # computing the eBLUPs, estimated genetic variance and C22 matrix
      gBLUP <- fit.cls$coefficients$random
      s.var <- summary(fit.cls)$varcomp['giv(plantid).giv','component']
      C22   <- predict(fit.cls, classify="giv(plantid)", only="giv(plantid)", vcov=T)$pred$vcov
```

```
    # removing stuff from memory
      rm(fit,fit.cls,ourdata,plantid)
      rm(inv.toyG)

## third-stage -- heritability and predictive accuracy estimation
  # preparing the matrices and auxiliary variables as in the paper notation
    G      <-toyG
    n      <-dim(G)[1]
    G.tilde<-G*s.var
    R.tilde<-solve(diag(n)*(wsmith))
    rm(toyG)

  # computing heritability and predictive accuracy via METHOD 5
    V    <-G.tilde+R.tilde
    P    <-(1/(n-1))*(diag(n)-matrix(1,n,n)/n)
    one  <-as.matrix(rep(1,n))
    Q    <-diag(n)-one %*% solve(t(one)%*%solve(V)%*%one) %*% t(one) %*% solve(V)
    C    <-G.tilde%*%solve(V)%*%Q

    PA.est.m5<-tr(P%*%C%*%G.tilde)/sqrt(tr(P%*%G.tilde)*tr(t(C)%*%P%*%C%*%V))
    H2.est.m5<-PA.est.m5^2
    rm(V,P,Q,C,one)

  # computing reliability and predictive accuracy via METHOD 7
    v1<-G.tilde
    v2<-G.tilde-C22
    rho2<-vector()
    for(j in 1:n){rho2[j]<-(v2[j,j])^2/(v1[j,j]*v2[j,j])}
    rm(j,v1,v2)

    RL.est.m7       <-mean(rho2)
    PA.est.m7       <-mean(sapply(rho2,sqrt))
    rm(rho2)

  # computing heritability via OAKEY's METHOD
    D     <-diag(n)-solve(G.tilde)%*%C22
    eival <-eigen(D)$values
    s     <-length(eival[eival<0.0001])

    H2.OAKEY<-tr(D)/(n-s)
    rm(D,eival,s,G.tilde,R.tilde)

    rm(n,w,wsmith)
    rm(G,C22)

  # printing out the results
    cbind(t(stage1.vars), s.var)
    cbind(H2.est.m5,H2.OAKEY,PA.est.m5,PA.est.m7)
```
